# Barriers to and Facilitators of Compliance with Clinic-Based Cervical Cancer Screening: Population-Based Cohort Study of Women Aged 23-60 Years

**DOI:** 10.1371/journal.pone.0128270

**Published:** 2015-05-26

**Authors:** Ellinor Östensson, Susanna Alder, K. Miriam Elfström, Karin Sundström, Niklas Zethraeus, Marc Arbyn, Sonia Andersson

**Affiliations:** 1 Department of Women’s and Children’s Health, Division of Obstetrics and Gynecology, Karolinska University Hospital-Solna, Karolinska Institutet, Stockholm, Sweden; 2 Department of Medical Epidemiology and Biostatistics, Karolinska Institutet, Stockholm, Sweden; 3 Department of Laboratory Medicine, Karolinska Institutet, Stockholm, Sweden; 4 Medical Management Centre (MMC), Department of Learning, Informatics, Management and Ethics (LIME), Karolinska Institutet, Stockholm, Sweden; 5 Belgian Cancer Centre/Unit of Cancer Epidemiology, Scientific Institute of Public Health, J Wytsmanstreet 14, B1050, Brussels, Belgium; Rudjer Boskovic Institute, CROATIA

## Abstract

**Objective:**

This study aims to identify possible barriers to and facilitators of cervical cancer screening by (a) estimating time and travel costs and other direct non-medical costs incurred in attending clinic-based cervical cancer screening, (b) investigating screening compliance and reasons for noncompliance, (c) determining women’s knowledge of human papillomavirus (HPV), its relationship to cervical cancer, and HPV and cervical cancer prevention, and (d) investigating correlates of HPV knowledge and screening compliance.

**Materials and Methods:**

1510 women attending the clinic-based cervical cancer screening program in Stockholm, Sweden were included. Data on sociodemographic characteristics, time and travel costs and other direct non-medical costs incurred in attending (e.g., indirect cost of time needed for the screening visit, transportation costs, child care costs, etc.), mode(s) of travel, time, distance, companion’s attendance, HPV knowledge, and screening compliance were obtained via self-administered questionnaire.

**Results:**

Few respondents had low socioeconomic status. Mean total time and travel costs and direct non-medical cost per attendance, including companion (if any) were €55.6. Over half (53%) of the respondents took time off work to attend screening (mean time 147 minutes). A large portion (44%) of the respondents were noncompliant (i.e., did not attend screening within 1 year of the initial invitation), 51% of whom stated difficulties in taking time off work. 64% of all respondents knew that HPV vaccination was available; only 34% knew it was important to continue to attend screening following vaccination. Age, education, and income were the most important correlates of HPV knowledge and compliance; and additional factors associated with compliance were time off work, accompanying companion and HPV knowledge.

**Conclusion:**

Time and travel costs and other direct non-medical costs for clinic-based screening can be considerable, may affect the cost-effectiveness of a screening program, and may constitute barriers to screening while HPV knowledge may facilitate compliance with screening.

## Introduction

Cervical cancer is major global health challenge and is the third most common cancer worldwide, although 80% of the burden is found in low-income countries that lack organized prevention programs [1]. Cytology-based organized cervical cancer screening programs have dramatically reduced cervical cancer incidence and mortality, since identification of precancerous lesions allows for successful treatment at early stages [2, 3]. The organized national cervical cancer screening program in Sweden is based on national recommendations and implemented at the county level. Within the organized screening program, midwives collect cervical smears at local out-patient clinics mainly during working hours (from 8 am to 5 pm), though opportunistic screening by gynecologists also occurs. All smears from both organized and opportunistic screening are registered in a national screening registry. The screening program invites women to participate every third year between ages 23 and 50 years and every fifth year between ages 51 and 60 years. Depending on County of residence, fees for a smear range between 0€ and ~22€. In Stockholm County (the location of the present study), smears are free of charge. Cervical cancer incidence in Sweden has fallen by 67% since the initiation of the organized screening program, although the rate of decline has stagnated somewhat in recent years [4]. Registry-based reports indicate that screening coverage in Sweden in 2012 was 78% for women aged 23–50 years for a 3.5-year time interval and 84% for women aged 51–60 for a 5.5-year time interval. Only around 5% of women never attend routine screening [5]. In 2012, the percentage of smears taken within the organized screening program was 67%. The percentage of women attending screening within 1 year of initial invitation was 64% nationally and 46% in Stockholm County. Response to initial screening invitation is an important indicator of access to preventive health care. According to a recent national audit of incident cervical cancer cases in Sweden, two of three women diagnosed were long-term non-attenders or attended the screening program only sporadically, which shows that women who do not attend according to national recommendations are at greater risk of developing cervical cancer [6].

Today, persistent infection with oncogenic human papillomavirus (HPV) types is known to precede the development of precancerous cervical lesions and cervical cancer; knowledge about the natural history of cervical disease has led to the development of two new methods of cervical cancer prevention: HPV vaccination and HPV testing for screening purposes [7]. The quadrivalent HPV vaccine was recently added to the organized school vaccination program in Sweden and is expected to reduce HPV prevalence, and consequently the incidence of cervical cancer in future generations. Primary HPV testing has proven more effective than conventional cytology in reducing both cervical cancer incidence and mortality [8–10]. The risk of cervical intraepithelial neoplasia grade 3 or cervical cancer is lower 5 years after a negative HPV test than 3 years after a negative cytology test, thus the HPV test has a higher longitudinal negative predictive value [11]. Changing from conventional cytology to HPV testing as a primary screening tool would lead to changes in screening program protocols: longer screening intervals and the use of cytology triage for HPV-positive women. This in turn would lead to the need for adjusted algorithms for the management of women who are HPV-positive but cytology-negative. In addition, the availability of self-sampling devices could make HPV testing more acceptable to those who are currently noncompliant with clinic-based screening due to a reluctance to undergo gynecological examination [12, 13].

According to the guidelines of the Swedish National Board of Health and Welfare [Socialstyrelsen], both clinical efficacy and cost-effectiveness must be confirmed before policy-makers can introduce new population-based screening programs or modify existing programs. Convention holds that cost-effectiveness should be assessed from a societal perspective, i.e., including all costs imposed on society [14]. Given the crucial role of screening compliance to achieve clinical effectiveness and cost-effectiveness in any screening program, identifying potential barriers to screening is especially important.

Prior research has shown that the use of healthcare services varies inversely with cost incurred or price paid, which is why it is important to offer either low-cost or free screening services [15–17]. Even when screening services are free, loss of income is still possible if one has to take time of work to attend screening. Indeed, when time and travel costs and other direct non-medical costs are perceived to be high, some individuals may be deterred from preventive health services such as screening. Previous economic analyses of screening programs have mainly focused on direct medical costs. They have rarely addressed attendees’ indirect costs (i.e., costs corresponding to the time needed for the screening visit) or direct non-medical costs related to clinic-based screening (e.g., transportation costs to and from the clinic, child care costs (e.g., not publicly-financed child care), or parking fees) [18]. Investigating whether screening compliance is impacted by time and travel costs and other direct non-medical costs will strengthen our understanding of how these factors influence the adherence to clinical regimens.

Research has shown that screening compliance is associated with women’s knowledge of cervical cancer and their attitudes toward screening [19]. Moreover, even though HPV is one of the most common sexually transmitted infections worldwide, general knowledge of HPV is low [20–23]. If public health officials are aware of the level of HPV knowledge in the population eligible for screening, they can better provide consistent and tailored health education.

This study aims to identify possible barriers to and facilitators of cervical cancer screening by (a) estimating time and travel costs and other direct non-medical costs incurred by women attending the clinic-based organized cervical cancer screening program in Sweden, (b) investigating screening compliance and reasons for noncompliance, (c) determining women’s knowledge of HPV, its relationship to cervical cancer, and HPV and cervical cancer prevention, and (d) investigating correlates of HPV knowledge and screening compliance.

## Materials and Methods

### Study design, study sample, and data collection

This descriptive population-based study included 1510 women who attended clinic-based organized cervical cancer screening at women’s outpatient clinics in nine municipalities in Stockholm County, Sweden. Of the nine outpatient clinics initially contacted, six agreed to participate (the municipalities of Danderyd, Nacka, Sundbyberg, Stockholm City, and Värmdö) and three declined (the municipalities of Jakobsberg, Botkyrka, and Nynäshamn). Those that declined typically served a largely non-Swedish-speaking patient population. Their inclusion would have required translation of the study materials into several different languages, which was beyond the means of the current study. Data were collected from March 2013 to April 2014. In order to be eligible for the organized cervical cancer screening program in Sweden, women must be aged 23 to 60 years and must not have undergone recent opportunistic screening; therefore all of the women in our study sample meet these criteria. Women were invited to participate in the study at the clinic, and those who agreed were given questionnaires, which they completed in the waiting room. Of all invited women, around 2% declined participation, usually because of language difficulties or a general lack of interest in completing questionnaires. Almost no missing responses to individual items in the questionnaire occurred (between 0–0.5% per item), since women could ask clarifying questions of the researchers while they were answering the questionnaire.

The study was approved by the Ethical Review Board at Karolinska Institutet, Stockholm, Sweden, which gave approval to seek oral informed consent before handing out the self-administered questionnaire. Due to the approval from the committee, written informed consent was not sought, but all participating women gave oral informed consent to complete the questionnaire after hearing a description of the research project. We considered their handing in of the questionnaire as confirmation of this consent, and the questionnaire itself as a record of this consent.

### Questionnaire

The questionnaire was developed based on the recommendations of a working group on patient-reported costs [24] and was influenced by previous questionnaires from studies concerning knowledge and attitudes on HPV [20, 23, 25] (S1 File). The questionnaire was piloted on a group of women with different occupations and refined through a validation process. The first part of the questionnaire explored sociodemographic characteristics, including age, education level, marital and employment status, and gross annual income. Sociodemographic characteristics were categorized and compared with official statistics obtained from Eurostat at the population level [26].

The second part of the questionnaire addressed time and travel costs and other direct non-medical costs incurred due to screening attendance, including estimated distances to and from the clinic, travel time to and from the clinic, mode of transportation, estimated net transportation costs, fares, parking fees (for car users only), child care costs (i.e., out-of-pocket costs incurred by women attending screening that are not associated with publicly-financed child care), and activities forgone. This part of the questionnaire also contained questions about estimated time taken off from work to attend screening, whether the woman was accompanied and if so, the companion’s relationship to the woman, companion’s income and companion’s estimated time off work. To estimate total time required at the clinic for screening, we measured and summed the average wait time and the time required for the screening procedure at two clinics on four different occasions for a total of 197 women.

Data from the second part of the questionnaire were sufficient to estimate time and travel costs, and other direct non-medical direct costs [27]. All costs are expressed using 2012 prices, which were converted from Swedish kronor (SEK) to Euro using the average exchange rate for 2012 (€1 = 8.7 SEK). Travel cost for car users was estimated at €0.45 per kilometer, based on a formula developed by the Swedish Automobile Association that estimated the gross cost per kilometer for three types new cars (2012) (priced €21 300, 26 400, and €33 900) and three cars from 2002 (priced €2 500, €6 800, and €8 900), which were assumed to be driven 15000 km annually. This formula generated an estimated cost per 10 km of €4.8, €5.8, €6,7 and €2.4, €3.2, €4, respectively, including estimates for fuel (€1.67 per liter gas/petrol, including 58% tax), taxes, depreciation, service, and repairs [28].

Travel costs for public transportation (i.e., bus, train, and subway) users were estimated at €0.13 per kilometer including taxes (25%). Estimates were calculated based on the cost of a 30-day public transportation pass (cost: €91.6), which makes up 70% of ticket sales for public transportation, and [29] mean distance traveled to and from work (i.e., 24 km) with public transportation [30]. Mean travel costs for taxi were estimated from the cost given by the respondents.

No private transportation costs were estimated for walkers and cyclists. Companions were assumed to incur the same mean travel costs to and from clinic as the respondents, except when they traveled together by car or taxi. Activities before attending screening were classified as work, formal education, leisure activity, and picking up/dropping off children at school or child care. Child care incurred costs while attending screening were estimated using the cost reported by the respondents.

When respondents or companions had taken time off work, the value of this time was estimated at €29.9 per hour using the Swedish gross wage rate (i.e., average yearly gross earnings of €53 985/1808 working hours). The average gross wage rate was defined as income from employment, self-employment, pension, sick pay, and other taxable income plus social security contributions (of 31.42%) for all occupational groups in both the public and private sector, adjusted for number of employees in the labor force in 2012 [31]. When respondents or companions stated that the respondent did not take time off work, time was costed at a rate of €12.38 per hour, which is a value of non-working time used when assessing Swedish public transportation projects and reflects average hourly take-home pay [32].

The third part of the questionnaire explored knowledge of the purpose of cervical cancer screening, HPV knowledge, screening compliance (which was defined as a woman stating that she usually attended screening within 1 year of the first invitation), and reasons for noncompliance. HPV knowledge was determined based on respondents’ agreement with 17 facts about HPV, its relationship to cervical cancer and preventive methods. Three responses were possible: “yes” (correct answer meaning agreement with the fact), “no” (incorrect answer meaning disagreement with the fact), or “don't know” (counted as an incorrect answer). The participants were to reply to these facts based on the knowledge they possessed prior to receiving the questionnaire.

### Statistical analyses

Prior to study initiation, we conducted a power calculation and determined that a total study sample of 1500 respondents would ensure a power of 0.976 to detect a 10% difference in the proportion of women from different age groups answering “yes” to the facts about HPV and its relationship to cervical cancer and preventive methods. Data were entered into the Statistical Package for Social Science (SPSS software version 21). Descriptive statistics including frequency distribution of variables and mean scores were obtained. To test internal reliability, Cronbach’s alpha coefficient was measured and an alpha ≥0.70 was considered to be satisfactory. When data were missing, percentages were recalculated to include only those who responded. The difference between mean transport costs by mode, travel time, and distance traveled was analyzed using one-way analysis of variance at 5%, with a Bonferroni correction. The Z-test was used to investigate whether sociodemographic characteristics differed significantly regarding any single (categorical) characteristic among respondents compared with the general female population in Sweden. We also used the Z-test to investigate whether HPV knowledge differed significantly between age groups.

A binomial logistic regression model was used to identify correlates of HPV knowledge and screening compliance. For both knowledge and compliance questions, we assigned a score of 1 to “yes” responses and a value of 0 “no” or “don’t know” responses. Correlates of HPV knowledge were determined using a dichotomous dependent variable based on the median (i.e., ≥5 or <5) HPV knowledge score. We performed a manual backwards step-wise procedure to determine the remaining variables in the model; non-significant variables (p <.05) were excluded. Variables were retained if their removal altered the width of the confidence intervals (CIs) of the remaining variables by more than 10%.

Qualitative data obtained from individual answers were used to support the quantitative data.

## Results

### Sociodemographic characteristics of the respondents

The sociodemographic characteristics of the 1510 respondents are presented in Table 1. A comparison with the general female population in Sweden, Stockholm County, and the municipalities of Nacka, Värmdö, Stockholm City, and Sundbyberg, showed that a significantly higher proportion (p <.01) of respondents had an education level above high school and there was a significantly greater proportion (p <.01) of respondents with a higher gross annual income (S1, S2 and S3 Tables).

**Table 1:** Sociodemographic characteristics of 1510 women attending the clinic-based cervical cancer screening program in Sweden.

Parameter	n, (%)
Age (years)	
≤24	109, (7.2)
25–29	198, (13)
30–34	297, (19.7)
35–39	236, (15.6)
40–44	244, (16.2)
45–49	219, (14.5)
50–54	108, (7.2)
≥55	99, (6.6)
Education level	
<High school	58, (3.9)
High school or equal	496, (32.8)
>High school	956, (63.3)
Marital status	
Married	790, (52.3)
Cohabitation	418, (27.1)
Single	287, (19)
Widow	13, (.9)
Together living apart	2, (.1)
Employment status	
Employed	1184, (78.5)
Self-employed	128, (8.5)
Student	117, (7.7)
Unemployed	45, (3)
Sick leave and retired	36, (2.4)
Working time	
Full time (≥100%)	1210, (80)
Part time (≥75%-99%)	166,(20)
Gross annual income (€)	
<13 783	139, (9.2)
13784–27 568	202, (13.4)
27569–41 353	477, (31.6)
41 354–55 137	339, (22.5)
55138–68 922	199, (13.2)
68923–82 707	81, (5.4)
82708–96 492	37, (2.5)
96493–110 276	16, (1.1)
≥110277	20, (1.3)

n = number.

### Travel characteristics, time and travel costs and other direct non-medical costs

Public transportation (29.7%), was the most common mode of transportation used by respondents, closely followed by car (27.8%) and walking (27.2%) (Table 2). A majority of the respondents paid for public transportation with a 30-day pass (628 women); 40 women paid with a zone ticket (cost range: €4-€6). Most respondents came to the clinic directly from their homes (85.5%), while 12.1% came from their workplaces. Following screening, more than half (59.6%) went to work, while 28.3% returned home and 4.2% traveled to their educational institution. Mean distance traveled to the clinic was 6.4 km and from the clinic was 11.4 km, yielding a mean total distance traveled of 17.8 km. Estimated average wait time was 10 minutes and screening procedure time was 13 minutes, for a total time of 23 minutes per screening visit. Average travel time was 18 minutes to the clinic and 26 minutes from the clinic (excluding time for other errands on the way), yielding a total travel time of 44 minutes (Table 3). The differences between mean travel time, distance traveled and travel costs by mode of transportation were significant in all comparisons.

**Table 2:** Travel characteristics of 1510 women attending the clinic-based cervical cancer screening program in Sweden.

Parameter	n, (%)
Point of departure to outpatient clinic	
Home	1290, (85.5)
Work	183, (12.1)
Educational institution	11, (.7)
Other	26 (1.7)
Activities before attending screening	471, (31.2)
Work	40, (2.6)
Formal education	13, (.9)
Leisure activity	131, (8.7)
Picking up/dropping off children at school or child care	287, (19)
Mode of transportation to outpatient clinic	
Car	420, (27.8)
Car as a passenger	65, (4.3)
Public transportation^a^	448, (29.7)
Bicycle	162, (10.7)
Walking	410, (27.2)
Taxi	5, (.3)
For car users, parking fee paid	76, (18.1)
Destination from outpatient clinic	
Home	428, (28.3)
Work	899, (59.6)
Educational institution	63, (4.2)
Other	120, (7.9)
Mode of transportation from outpatient clinic	
Car	406, (26.9)
Car as a passenger	23, (1.5)
Public transportation^a^	618, (40.9)
Bicycle	139, (9.2)
Walking	246, (16.3)
Taxi	2, (.1)
Women was accompanied	182, (12.)
Partner	131, (8.7)
Relative	33, (2.1)
Friend or other	18, (1.2)

n = number.

^a^Includes bus, train and subway.

**Table 3:** Time and travel costs, direct non-medical costs and other related variables for 1510 women attending the clinic-based cervical cancer screening program in Sweden.

Mean total time elapsed (minutes) per attendance^a^		23
Mean total distance traveled round trip (km)		17.8
Mean total travel time round trip (minutes)		44
Mean transport cost, subjects only, by mode (€)	*To clinic*	*From clinic*
Car	2.9	5.1
Public transportation^b^	0.8	1.5
Taxi	20.1	31.8
Total	1.9	2.9
Proportion of respondents taking time off work (n,%)		807, 53.4
Mean total time off work (minutes)^c^		147
Proportion of companions taking time off work (n,%)		108, 59.3
Mean total time off work for companion (minutes)^d^		113
Mean cost for production loss per attendance (€)		73.3
Mean cost for production loss per companion attending (€)		56.2
Proportion of child care (n,%)		42, 2.8
Mean total time for paid child care (minutes)^e^		168
Mean cost for paid child care per attendance (€)^f^		78.4
Mean total time for unpaid child care per attendance (minutes)^g^		171
Mean cost for unpaid child care during leisure time per attendance (€)^h^		35.4
Mean cost for parking fee (€)^i^		2.8
Mean total cost per attendance (€)		50.8
Mean total cost per companion (€)		43.8
Mean total cost per attendance including companion, if any (€)		55.6

n = number.

^a^Based on data on average time for medical procedure and wait time from a total of 348 attendances in two outpatient clinics at four occasions respectively.

^b^Includes bus, train and subway.

^c^A total of 1980 hours off work were estimated based on 807 women (1980/807 = 147 minutes).

^d^A total of 203 hours off work were estimated based on 108 individuals accompanying the women(203/108 = 113 minutes).

^e^A total of 98 hours were estimated as child care for 35 women (98/35 = 168 minutes).

^f^A cost of €28 per hour were calculated on an estimated total cost of €2740 paid for 98 hours child care (28x2.8 = €78.4).

^g^A total of 20 hours were estimated as unpaid child care for 7 women (20/7 = 171 minutes).

^h^A total cost of €247.6 for unpaid child care were estimated based on €12.38 per hour (i.e., cost for leisure time). Mean cost were then calculated on 7 women attending screening (247.6/7 = €35.4).

^i^A total of 85 women paid parking fees estimated to a total of €238.5 (238.5/85 = €2.8).

Over half the respondents (53.4%) reported taking time off work to attend screening; mean time 147 minutes (Table 3). Among all respondents, 12% were accompanied by a companion, the majority by their partners. Among all companions, 59.3% had taken time off work (mean time: 113 minutes). Among all responders 42 women (2.8%) had arranged child care. For 35 women, this was an out-of-pocket cost incurred when attending screening (mean time: 168 minutes, estimated cost: €78.4). Mean total travel costs to the clinic amounted to €1.9 and from the clinic amounted to €2.9. Mean total costs (time and travel costs and other direct non-medical costs) per screening attendance amounted to €50.8, and the mean total cost per attendance including companion (if any) was €55.6.

### HPV knowledge and knowledge of cervical cancer screening

In all, 68.9% of respondents knew that screening was meant to prevent cervical cancer, while 31.1% believed that cytology testing was used to screen for all gynecological cancers. Almost half (49.5%) of the respondents were satisfied with the information provided in the invitation letter while 27.5% were unsatisfied and others were either partly satisfied or answered “don’t know”. Compared with respondents aged 30–49 years, a significantly higher proportion of respondents aged 29 years or younger knew that HPV is sexually transmitted (51.0% vs 39.1%), that both men and women can be infected (30.4% vs 23.5%) and that HPV is most common among young adults (36.9% vs 27.4%). Respondents aged 29 years or younger also knew that HPV infections often had no symptoms (40.5% vs 35.1%); that persistent HPV infection may lead to cervical cytological abnormalities (54.0% vs 48.8%); and were more aware that HPV can cause genital warts (24.3% vs 22.2%). Of all respondents, 63.7% knew that an HPV vaccine is available, but fewer were aware that the vaccine is most effective if administered before sexual debut (i.e., when a girl is HPV naïve) (40.7%) and that the vaccine does not protect against all HPV types, therefore making it important to continue to attend screening (33.9%). However, only 6.7% believed they had a good knowledge of HPV and cervical cancer, and only 16.1% felt they had a good knowledge of HPV and cervical cancer prevention (Table 4). A majority of women (63.2%) expressed a desire to learn more about HPV infection, risk factors and prevention from a midwife or physician, while 52.1% wanted additional information from brochures.

**Table 4:** Human papillomavirus (HPV) knowledge among 1510 women attending the clinic-based cervical cancer screening program in Sweden.

		Age group			
	Facts about HPV and replies	≤29 y (%)	30–49 y (%)	50-60y (%)	≤60 y (%)
		n = 307	n = 996	n = 207	n = 1510
A)	There are around 200 known types of HPV types.				
	*Yes*	23.1	10.5	9.7	12.9
	*No*	22.7	32.3	30.9	30.2
	*Don’t know*	54.2	57.2	59.4	56.9
B)	Among all these types, around 40 can infect the genital area.				
	*Yes*	23.6	13.8	10.6	15.4
	*No*	26.2	31.8	33.3	30.9
	*Don’t know*	50.2	54.4	56	53.7
C)	HPV is sexually transmitted.				
	*Yes*	51	39.1	32.4	40.6
	*No*	22.1	31.1	27.5	28.8
	*Don’t know*	26.9	29.8	40.1	30.7
D)	Both men and women can get infected.				
	*Yes*	30.4	23.5	21.7	24.7
	*No*	30.4	35	30.4	33.4
	*Don’t know*	39.2	41.4	47.8	41.9
E)	HPV is most common among young adults but can occur at all ages.				
	*Yes*	36.9	27.4	21.3	28.5
	*No*	28.8	33.1	32.4	32.1
	*Don’t know*	34.3	39.5	46.4	39.4
F)	Most HPV infections have no symptoms.				
	*Yes*	40.5	35.1	25.1	34.8
	*No*	23.9	28.9	30	28
	*Don’t know*	35.6	36	44.9	37.2
G)	There is no treatment for HPV infection.				
	*Yes*	24.3	14.7	14.5	16.6
	*No*	35.6	39	38.2	38.2
	*Don’t know*	40.1	46.3	47.3	45.2
H)	Most of the HPV infections are transient				
	*Yes*	21	15.8	11.1	16.2
	*No*	39.8	38.1	43	38.9
	*Don’t know*	39.8	46.1	45.9	44.9
I)	A persistent HPV infection may cause cytological abnormalities on cervix.^a^				
	*Yes*	54	48.8	41.1	48.8
	*No*	17.2	22.6	18.8	21
	*Don’t know*	28.7	28.6	40.1	30.3
J)	After many years, cytological abnormalities may lead to cervical cancer.^b^				
	*Yes*	68.9	68.9	59.9	67.7
	*No*	10.4	12.7	9.2	11.7
	*Don’t know*	20.7	18.4	30.9	20.6
K)	There are around 20 different HPV types that can cause cervical cancer. Of these, HPV 16 and HPV 18 cause almost 70% of all cervical cancer cases.				
	*Yes*	19.7	12.6	14.5	14.3
	*No*	23.9	30.6	26.1	28.6
	*Don’t know*	56.3	56.8	59.4	57.1
L)	Other types of HPV can cause genital warts among both men and women.^c^				
	*Yes*	24.3	22.2	18.8	22.2
	*No*	29.4	32.7	30.4	31.7
	*Don’t know*	46.3	45.1	50.7	46.1
M)	Vaccination is one way to protect against HPV infection that can cause abnormal cervical smears and in some cases cervical cancer. ^d^				
	*Yes*	66	70.1	55.6	63.7
	*No*	12.3	11.9	16.9	12.6
	*Don’t know*	21.7	18	27.5	20.1
N)	Vaccination is most effective before sexual debuts’^e^				
	*Yes*	42.4	40.3	40.1	40.7
	*No*	24.3	30.1	24.6	28.1
	*Don’t know*	33.3	29.6	35.3	31.1
O)	The vaccine only protects against the most common HPV types that cause cervical cancer and it is therefore important to continue to attend screening for cervical cancer. ^f^				
	*Yes*	34.6	34.2	31.4	33.9
	*No*	35.0	36.7	31.4	35.6
	*Don’t know*	30.4	29.1	37.2	30.5
P)	I believe I have good knowledge of HPV and cervical cancer. ^g^				
	*Yes*	8.1	6.8	3.9	6.7
	*No*	83.2	85.8	87.4	85.5
	*Don’t know*	8.7	7.4	8.7	7.8
Q)	I believe that I have good knowledge in how to prevent HPV infection and cervical cancer. ^h^				
	*Yes*	15.6	17.6	9.7	16.1
	*No*	74.4	75.2	77.8	75.3
	*Don’t know*	10.1	7.2	12.5	8.5

y = years.

n = number.

All differences between groups are significant at *p<* .05 except following:

^a^ *p*-value .13 between women ≤29 y and women 30–49 y.

^b^ *p*-value .94 between women ≤29 y and women 30–49 y.

^c^ *p*-value .43 between women ≤29 y and women 30–49 y, .12 between women ≤29 y and women 50–60 y, .22 between women 30–49 y and women 50–60 y.

^d^ *p*-value .19 between women ≤29 y and women 30–49 y.

^e^ *p*-value .52 between women ≤29 y and women 30–49 y, .61 between women ≤29 y and women 30–49 y,. 97 between women 30–49 y and women 50–60 y.

^f^ *p*-value .9 between women ≤29 y and women 30–49 y, .46 between women ≤29 y and women 50–60 y, .37 between women 30–49 y and women 50–60 y.

^g^ *p*-value .41 between women ≤29 y and women 30–49 y, .052 between women ≤29 y and women 50–60 y, .12 between women 30–49 y and women 50–60 y.

^h^ *p*-value .36 between women ≤29 y and women 30–49, .061 between women ≤29 y and women 50–60 y.

### Screening compliance

Among all respondents, 44.3% said that they were noncompliant. The reasons given for non-compliance were inability to take time off work (51.4%), too busy (32.9%) and other reasons (15.8%) (Table 5), which included things such as fear of gynecological examination, negative experiences at previous screening visits, postponement due to menstrual period, and pregnancy at the time of invitation.

**Table 5:** Screening compliance for 1510 women attending the clinic-based cervical cancer screening program in Sweden.

		Age group		
Parameter	≤29 y	30–49 y	50–60 y	23–60 y
	(%)	(%)	(%)	(%)
Compliance to screening usually within 1 year after invitation	n = 309	n = 994	n = 207	n = 1510
*Yes*	42.1	54.6	45.4	50.5
*No*	39.5	42.6	49.8	44.3
*Don’t know*	18.4	2.2	5.3	6.3
Reason for non-compliance	n = 151	n = 423	n = 102	n = 647
*Too busy*	40.4	30	35.2	32.9
*Can´t take time off work*	30.5	57.7	47.6	51.4
*Other*	29.1	12.3	17.2	15.8

y = years. n = number. All differences between groups are significant at *p <*.05

### Correlates of HPV knowledge and screening compliance

The most important correlates of screening compliance were age, education, income, accompanying companion and time off work to attend screening. For HPV knowledge, these factors were age, education and income (Table 6).

**Table 6:** Correlates of human papillomavirus (HPV) knowledge and screening compliance for 1510 women attending the clinic-based cervical cancer screening program in Sweden.

	HPV knowledge^a^	Screening compliance^a^
Parameters	OR (95% CI)	OR (95% CI)
Age (years)		
≥55	Reference	Reference
≤24	1.9 (1.01-3-55)	.43 (.23-.82)
25–29	1.89 (1.09–3.28)	.89 (.51–1.53)
30–34	1.52 (.91–2.55)	1.37 (.83–2.27)
35–39	1.32 (.78–2.23)	1.1 (.66–1.86)
40–44	1.39 (.83–2.35)	1.66 (.99–2.77)
45–49	1.98 (1.17–3.37)	2.1 (1.24–3.53)
50–54	2.35 (1.28–4.30)	1.23(.68–2.22)
Education level		
<High school	Reference	Reference
High school or equal	3.23(1.56–6.66)	1.72(.92–3.19)
>High school	8.67 (4.19–17.93)	1.32(.70–2.46)
Total annual income (€)		
<13 783	Reference	Reference
13784–27 568	1.03 (.64–1.65)	0.8 (.49–1.28)
27569–41 353	.66 (.43–1.02)	1.76 (1.13–2.75)
41 354–55 137	.54 (.34-.87)	1.45 (.90–2.33)
55138–68 922	.57 (.34-.94)	1.62 (.95–2.74)
68923–82 707	.28 (.15-.53)	1.07 (.56–2.05)
82708–96 492	1.54 (.66–3.62)	1.26 (.56–2.84)
96493–110 276	.23 (.07-.74)	2.43 (.74–8.02)
≥110277	.38 (.14–1.03)	1.32 (.46–3.77)
No time off work		Reference
1 hour off work		.62 (0.43-.89)
2 hours off work		.46 (0.34-.62)
3 hours off work		.27 (.18-.39)
>4 hours off work		.40 (.27-.60)
No companion		Reference
Companion		.51 (.36–0.71)
No HPV knowledge*		Reference
HPV knowledge		2.4(1.91–3.02)

OR = odds ratio. CI = confidence interval.

^a^Two separate models were generated for the cohort after manual backward stepwise selection excluding non-significant variables. Therefore, not all variables were applicable in both models.

*Correlates of knowledge were determined using a dichotomous dependent variable based on the median (i.e.,≥ 5 or <5) HPV knowledge score. Therefore, “knowledge of HPV” is referred to ≥5 on the HPV knowledge score and “No knowledge of HPV” referred to <5 on the HPV knowledge score.

## Discussion

The most important findings that emerged from this study were that time and travel costs and other direct non-medical costs incurred in attending clinic-based cervical cancer screening can be considerable, may affect the cost-effectiveness of a screening program and may constitute barriers to screening while HPV knowledge may facilitate compliance with screening. Screening compliance was significantly associated with age, income, time off work, accompanied by a companion, and HPV knowledge. Women that did not respond that they took time off work were more likely to attended screening within 1 year of initial invitation. Our earlier study calculated the direct cost of an initial visit to undergo cytological screening, performed in a hospital by a midwife, at about €72 in 2008 values, which translated to €82 in 2012 [33]. This implies that the estimated time and travel costs and other direct non-medical costs of €55.6 represent 68% of direct medical costs and are therefore sizable compared with the cost of the medical procedure itself.

Other studies have confirmed our findings that these costs are sizeable in relation to direct medical costs (between 20–90%) [34, 35]. Mean time and travel costs per attendance in this study were somewhat higher than those found in another study from the United Kingdom [36]. This difference may be explained by a longer mean time for the screening visits in our study. In addition, unpaid time in the study from the United Kingdom was valued at approximately half of our estimate. Different opinions exist over how to assign value to unpaid time [37–39]. One opinion is that unpaid time should be valued at zero cost. An alternative opinion is that it should be more valuable than paid time since employers are normally obliged to pay a disproportional rate to encourage employees to give up their leisure time. However, when the value of leisure time from the aforementioned study (£3.68/hour expressed in 2002 values translated in to €6.43 in 2012) [36] was applied in our calculations, excluding extra costs for time taken off work above and beyond the estimated mean travel time and screening visit time (67 minutes), we reached cost estimates similar to those reported in our study. This highlights the importance of context-specific estimates in the overall cost estimation process.

Women who attended screening with a companion and those who arranged for paid child care had substantially higher mean time and travel costs and other direct non-medical costs. Our data cannot explain the choice to attend screening with a companion, nor to arrange for child care outside the publicly-financed system, but these factors have a major impact on cost. Further research on choice of companion and need for or child care should be initiated.

Mean distance traveled and travel time in our study corresponds to previous reports on travel to work within Stockholm county (i.e., 12 km and 29 minutes) [30]. Proximity to a clinic is also an important consideration when estimating costs for the entire country of Sweden, as close proximity enables women to attend using a mode of transport with zero cost (such as cycling or walking). In our study, a high percentage (38%) of respondents walked or cycled to the clinic. Longer distances traveled resulted in higher time costs for the respondents and their companions, and for child care (if any). This study only includes data for the initial screening, which takes place at outpatient clinics. Potential follow-up visits usually take place at hospitals, which are more centralized and therefore farther away, entailing time and travel costs that are higher than those presented in this study. Therefore, other studies might include different time and travel costs and other direct non-medical costs based on variables such as proximity, density, net cash outlay and value of unpaid time.

In Sweden today, health care is predominantly financed by taxes. However for preventive health care such as screening, a copayment is required; an out-of-pocket expense that ranges from zero to about €22, depending on the county of residence. The importance of this cost in relation to screening compliance was shown in 2003, when a fee of €14 per screening visit was introduced in Stockholm, which resulted in a 23% decline in attendance and a subsequent rise in attendance again when the fee was removed [40]. However, even when screening is offered at no charge (as in Stockholm where this study was performed) women may still consider the time and travel costs and other direct non-medical costs required, which could deter them from attending screening. In our study, 44% of respondents stated that they were unable to attend clinic-based organized screening within 1 year of the initial invitation, of whom 51% stated that the reason for this was that they could not take time off from work to attend screening. This is in line with previous reports based on registry data (46% compliance with initial invitation within 1 year in Stockholm County, Sweden in 2012) [5] and suggests that women take such issues under consideration before attending screening. Being “too busy” was the second major reason (33%), which has been confirmed as a reason for non-attendance across age groups in previous research [41]. However it should be noted that women who stated that they typically did not attend within screening 1 year of receiving the invitation would still be classified as compliant with recommendations after the screening visit they attended for this study. They should thus not necessarily be placed in the same category as women who never attend screening or have very long periods of non-compliance. Indeed, a qualitative study on women attending cervical cancer screening in Stockholm found that many women desired increased flexibility in when and where to attend screening in order to facilitate their attendance [42]. Our study results imply that time taken off work for attending screening is an important factor that may influence compliance with cervical cancer screening.

The increased flexibility that could be offered through self-testing, which could be performed during leisure time at home, where time and travel costs are small, could possibly be used to influence compliance with cervical cancer screening among women that have difficulties in taking time off from work to attend screening, as well as to increase participation among women who are currently underrepresented in the screening program, specifically women with a lower education level and those in lower income brackets [12, 13]. Another important target group which was not reached in this study is immigrants, which constitute around 16% of the Swedish population, and have been shown to have a lower participation to cervical cancer screening [31, 43].

However, it must be emphasized that the relatively high direct non-medical costs and indirect costs presented in this study cannot definitively determine the cost-effectiveness of self-sampling-based screening over clinic-based screening. Such confirmation would require a full-scale formal assessment of all benefits and costs accrued by the both screening methods. The women in this study had a low to moderate knowledge of HPV and its relationship to genital warts and cervical cancer, which is consistent with past studies in Europe and the United States [20, 23, 44, 45]. The most important finding was that younger women were more knowledgeable about HPV than older women. However, there was no statistically significant difference between the age groups regarding knowledge of HPV prevention, including HPV vaccination. This may imply that the introduction of HPV vaccination into the school vaccination program has impacted the level of knowledge about HPV prevention among younger and older age groups alike. Previous targeted information and commercial campaigns carried out prior to this study may also have increased women’s knowledge of HPV, but to what extent remains unclear. In our study, the majority of women wanted to learn more about HPV and preventive methods through a personal counseling session with a midwife or physician. Previous research has shown that differences in HPV knowledge decrease after any type of intervention or public campaign to inform women, and specifically that brief personal counseling sessions are effective communication tools [46–50].

There is uncertainty as to whether HPV-vaccinated women will reduce their attendance to cervical cancer screening due to altered screening attitudes after vaccination [51–56]. Previous studies have suggested that early screening habits are indicative of screening attendance later in life [53–55]. In our study, only 34% of the respondents were aware that screening compliance is still important after HPV vaccination, illustrating the need to disseminate correct information on the function of cervical cancer screening in the era of HPV vaccination.

Since non-medical direct and indirect costs have been somewhat overlooked in the literature to-date, this study contributes new data that can be used in future cost-effectiveness studies evaluating interventions from the broadest societal perspective. We are also contributing information about the association between screening and HPV knowledge and compliance. We were able to investigate these issues in six population-based screening clinics; we obtained almost complete participation and had no missing responses in the questionnaire, which entails that our data collection was of high quality.

Several limitations to this study need to be acknowledged. Importantly, this study was performed in a county where there is no fee for a cervical smear. Thus, we could not ask women about the potential effect of fees on screening compliance. Furthermore, we only included women attending cervical cancer screening, and thus this study does not focus on women who never attend screening. Also, because of its non-randomized study design and the under-representation of women of low socioeconomic status and immigrant women, selection bias cannot be ruled out, which would preclude generalizability to women in lower socioeconomic groups. However it should be noted that the level of women with low socioeconomic status in our study is consistent with that of prior studies on cervical cancer screening attendance, highlighting the challenge in capturing information on screening behavior, attitudes, knowledge and costs across socioeconomic groups [57]. Despite the over-representation of women with higher socioeconomic status in this study, it may not be unreasonable to assume that our observation of costs and time functioning as barriers to screening could apply to women with low socioeconomic status as well. Indeed, it is possible that the impact of time, travel costs, other direct non-medical costs, and knowledge of HPV might further aggravate the differences in usage of screening services across socioeconomic strata. Further research is needed to investigate this hypothesis among both Swedish-born women of lower socioeconomic status and immigrant women of differing socioeconomic status, including whether there is an effect associated with fees for smears in areas where such fees are charged. Further research on women who never attend screening is also needed.

## Conclusion

In countries with organized cervical cancer screening programs, equal access to these is an important objective. Our study showed that time required, travel costs and other direct non-medical costs for clinic-based screening can be considerable. This may affect the cost-effectiveness of a screening program, and may constitute barriers to attending cervical screening even when there is no direct fee for the woman. Also, women with knowledge of HPV were more likely to comply with screening recommendations. Since the effectiveness of cervical cancer screening programs depends on compliance; increased flexibility in screening set-up and enhanced public knowledge of HPV could potentially act as facilitators to screening compliance.

## Supporting Information

S1 File Questionnaire in English. (DOCX)

S1 Table Difference in socio-demographic variables. (DOCX)

S2 Table Difference in educational level and income distribution. (DOCX)

S3 Table Difference in educational level and income distribution. (DOCX)
