# Correction: Barriers to and Facilitators of Compliance with Clinic-Based Cervical Cancer Screening: Population-Based Cohort Study of Women Aged 23-60 Years

**DOI:** 10.1371/journal.pone.0135534

**Published:** 2015-08-06

**Authors:** Ellinor Östensson, Susanna Alder, K. Miriam Elfström, Karin Sundström, Niklas Zethraeus, Marc Arbyn, Sonia Andersson

There are errors in the Funding section of the published article. The correct funding information is as follows: Swedish Cancer Foundation (070623, CAN 2007/1044, 11 0544, CAN 2011/471) URL: www.cancerfonden.se, SA; Karolinska Institutet Cancer Strategic grants (5888/05-722) URL: www.ki.se, SA; Swedish Research Council (521-2008-2899) URL: www.vr.se, SA; Stockholm County Council (20130097) URL www.skl.se, SA. Marc Arbyn was supported by the seventh framework program of DG Research of the European Commission, through the COHEAHR Network (grant No 603019). The funders had no role in study design, data collection and analysis, decision to publish, or preparation of the manuscript.
