# Supplementary material for: Barriers to and Facilitators of Compliance with Clinic-Based Cervical Cancer Screening: Population-Based Cohort Study of Women Aged 23-60 Years
Source: PLoS One. 2015 May 26;10(5):e0128270. doi: 10.1371/journal.pone.0128270 (PMC4444356; doi:10.1371/journal.pone.0128270)
Supplement: S1 File — (DOCX) [file pone.0128270.s001.docx]

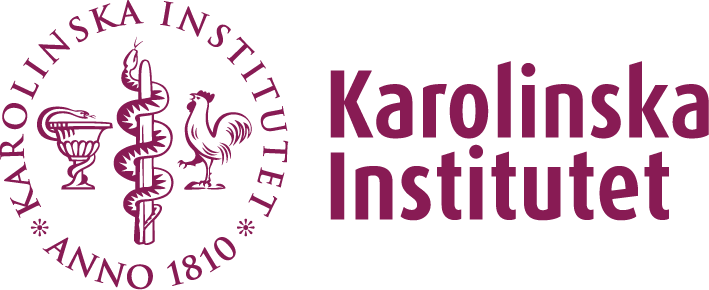


**This study is about the costs for time and travel related to screening with cytology testing and knowledge about human papillomavirus (HPV) infection and prevention.**

**Part 1** **Below are questions about your background, as well as costs for time and travel related to screening with cytology testing. *Please check the boxes for the answers that suit you best.***

| **No.** | **Questions** |
| --- | --- |
| **F101** | How old are you? …………….. |
| **F102** | **What is your marital status?**  1. Married/partnership 4. Widow  2. Cohabitating 5. Other (please specify)…………………  3. Single 999. Prefer not to answer |
| **F103** | **What is your main occupation?** ***Several answers are possible.***  1. Gainfully employed 5. Jobseeker  2. Self-employed 6. On sick leave  3. Student 7. Other (please specify)…………………  ……………………………………………......................................................... |
| **F104** | **How many hours do you usually work during a week (including overtime)? *Enter the percentage of time that you spend working/unemployed/studying, etc.***  Enter the number of whole hours:.......... (Only digits. Example: 40) |
| **F105** | **What is your monthly income before taxes?** **Income refers to salary, pension, student aid, compensation from insurance fund, income from own business or farm.**  1. SEK 1-10,000 5. SEK 41,000-50,000 9. SEK 81,000-90,000  2. SEK 11,000-20,000 6. SEK 51,000-60,000 10. SEK 91,000 or more  3. SEK 21,000-30,000 7. SEK 61,000-70,000  4. SEK 31,000-40,000 8. SEK 71,000-80,000 |
| **F106** | **What is your highest level of completed education?** ***Only one answer is possible***  1. Nine-year compulsory school 3. University/College  2. Upper secondary school 4. Other (please specify)…………………  ……………………………………………………………………….. |
| **F107** | **What means of transportation did you take to get here?** **If more than one, specify the main method.**  1. By foot 5. Local/regional bus  2. Bicycle 6. Tram/underground/subway  J  3. Car as driver 7. Regional train/commuter train  J    4. Car as passenger 8. Taxi  J  9. Other (please specify)…………………  ………………………………………………………………..  J |
| **F108** | **Where did you travel from to come to the clinic for screening?**  1. Own home 3. School/Training  2. Own job 4. Other (please specify)…………………  J  …………………………………………………………. |
| **F109** | **Did you do any other errands on your way here?**  1. Yes  2. No(**Go directly to F111)** |
| **F110** | **What was your errand on your way to the clinic?**  1. Work 6. Visit friends and family  2. School 7. Pick up/drop off child(ren)  3. Shopping 8. Pick up/drop off another person  4. Service (bank, post office, etc.) 9. Other private errand  5. Recreation/leisure activities |
| **F111** | **About how many minutes did it take you to travel to the clinic?** ***Exclude the time for any other errands***  …………..minutes |
| **F112** | **What is the approximate distance in kilometers between the town/city from which you traveled and the clinic?**  …………. kilometers |
| **F113** | **Where will you go after your visit to the clinic?**  1. Own home 5. School/Training  2. Own workplace 6. Other (please specify)…………………  J    ………………………………………………………….. |
| **F114** | **What means of transportation will you take to return to this location?** **If more than one, specify the main method.**  1. By foot 5. Local/regional bus  2. Bicycle 6. Tram/underground/subway  J  3. Car as driver 7. Regional train/commuter train  J    4. Car as passenger 8. Taxi  J  9. Other (please specify)…………………  ………………………………………………………………..  J |
| **F115** | **About how many minutes will it take you to return to that location after your visit to the clinic?**  ……………………minutes |
| **F116** | **About how many kilometers do you estimate that it is between the clinic and the place to which you will travel?**  …………. kilometers |
| **F117** | **Only answer F117-F119 if you traveled mainly by car.** **About how much do you estimate that you paid to travel here?** **Include the cost per kilometer by car.**  SEK …………….. |
| **F118** | **Did you pay a parking fee?**  1. Yes 2. No **– Go directly to F120** |
| **F119** | **About how much did you pay for parking?**  SEK ……………… |
| **F120** | **Only answer F120-F123 if you traveled mainly by public transportation.** **Do you have a travel pass (7 -day pass, 30-day pass, 1-year pass or similar)?**  1. Yes 2. No **– Go directly to F122** |
| **F121** | **How much did your travel pass cost?**  SEK ……………… |
| **F122** | **Did you pay for your trip with a zone ticket (ticket for short or individual trips)?**  1. Yes 2. No **– Go directly to F124** |
| **F123** | **How much did your zone ticket cost?**  SEK …………….. |
| **F124** | **Did you take time off from work to go for screening?**  1. Yes 2. No **– Go directly to F126** |
| **F125** | **How many hours did you take off from work?**  ……………hours |
| **F126** | **Did you need help from another person to come for screening today (such as child care or other support)?**  1. Yes 2. No **– Go directly to Part 2** |
| **F127** | **What is your relationship to this person?**  1. Partner 4. Purchased child care service-**Go directly to F131**  2. Relative 5. Other (please specify)…………………  3. Friend 999. Prefer not to answer |
| **F128** | **Did this person have to take time off from work?**  1. Yes 2. No 998. Don't know |
| **F129** | **About how many hours do you estimate that this person took off from work?**  ……………hours |
| **F130** | **What is this person’s monthly income before taxes?** **Income refers to salary, pension, student aid, compensation from insurance fund, income from own business or farm.**  1. SEK 1-10,000 5. SEK 41,000-50,000 9. SEK 81,000-90,000  2. SEK 11,000-20,000 6. SEK 51,000-60,000 10. SEK 91,000 or more  3. SEK 21,000-30,000 7. SEK 61,000-70,000  4. SEK 31,000-40,000 8. SEK 71,000-80,000 |
| **F131** | **Only answer F131-F132 if you traveled mainly by public transportation.** **For about how many hours did you hire child care?**  ……………hours |
| **F132** | **About how much did you pay for child care?**  SEK……………per hour 999. Prefer not to answer |

**Part 2.** **Below are a few questions about screening with cytology testing and factual statements about human papillomavirus (HPV).** ***Please check the box for the answer that suits you best.***

| **No.** | **Questions** |
| --- | --- |
| **F201** | **What type of gynecological cancer do you think cytology testing is meant to prevent?**  1. Cervical cancer 3. Ovarian cancer  2. Uterine cancer 4. All gynecological cancer |
| **F202** | **Do you think you received sufficient information about cytology testing in the invitation?**  1. Yes, completely **– Go directly to F204** 3. No  2. Partly 998. Don't know |
| **F203** | **What information do you feel was missing in the invitation?** **……………………………………………………………………………………………………………………………………**  **…………………………………………………………………………………………………………………………………..**  **…………………………………………………………………………………………………………………………………..**  **…………………………………………………………………………………………………………………………………..**  **…………………………………………………………………………………………………………………………………..** |
| **F204** | **Do you usually go for cytology testing within one year of the time you receive the initial invitation?**  1. Yes – **Go directly to F206** 2. No 998. Don’t know |
| **F205** | **Why don’t you usually go for cytology testing within one year of the time you receive the initial invitation?**  1. Too busy  2. Can´t take time off work  3. Other (please specify)…………………………………………………………………………………..  ..….. …………………………………………………………………...…………………………………………………..  ……………………………………………………………………………………………………………………………….. ……………………………………………………………………………………………………………………………….. ………………………………………………………………………………………………………………………………… ………………………………………………………………………………………………………………………………… …………………………………………………………………………………………………………………………………. ………………………………………………………………………………………………………………………………….. …………………………………………………………………………………………………………………………………..  …………………………………………………………………………………………………………………………………… |
| **No.** | **Below are factual statements about HPV.** **Indicate whether you were aware of each factual statement before you participated in this study with the response options:** **Yes, No, or Don’t know.** ***Try to be as honest as possible in your responses.*** |
| **F206** | **There are around 200 known types of HPV.**  1. Yes 2. No 3. Don´t know |
| **F207** | **Among all these types, around 40 can infect the genital area.**  1. Yes 2. No 3. Don´t know |
| **F208** | **HPV is sexually transmitted.**  1. Yes 2. No 3. Don´t know |
| **F209** | **Both men and women can get infected.**  1. Yes 2. No 3. Don´t know |
| **F210** | **HPV is most common among young adults but can occur at all ages.**  1. Yes 2. No 3. Don´t know |
| **F211** | **Most HPV infections have no symptoms.**  1. Yes 2. No 3. Don´t know |
| **F212** | **There is no treatment for HPV infection.**  1. Yes 2. No 3. Don´t know |
| **F213** | **Most HPV infections are transient.**  1. Yes 2. No 3. Don´t know |
| **F214** | **A persistent HPV infection may cause cytological abnormalities on the cervix.**  1. Yes 2. No 3. Don´t know |
| **F215** | **After many years, cytological abnormalities may lead to cervical cancer.**  1. Yes 2. No 3. Don´t know |
| **F216** | **There are around 20 different HPV types that can cause cervical cancer. Of these, HPV 16 and HPV 18 cause almost 70% of all cervical cancer cases.**  1. Yes 2. No 3. Don´t know |
| **F217** | **Other types of HPV can cause genital warts among both men and women.**  1. Yes 2. No 3. Don´t know |
| **F218** | **Vaccination is one way to protect against HPV infection that can cause abnormal cervical smears and in some cases cervical cancer.**  1. Yes 2. No 3. Don´t know |
| **F219** | **Vaccination is most effective before sexual debut.**  1. Yes 2. No 3. Don´t know |
| **F220** | **The vaccine only protects against the most common HPV types that cause cervical cancer and it is therefore important to continue to attend screening for cervical cancer.**  1. Yes 2. No 3. Don´t know |
| **No.** | **Below are some questions regarding your perception of your HPV knowledge before you participated in this study. *Please check the box for the answer that suits best.*** |
| **F221** | **I believe I had good knowledge of HPV and cervical cancer.**  1. Yes 2. No 3. Don´t know |
| **F222** | **I believe that I had good knowledge about how to prevent HPV infection and cervical cancer.**  1. Yes 2. No 3. Don´t know |
| **F223** | **If you feel that you need more information about HPV, cervical cancer, and preventive methods, how would you like to be informed?** ***Several answers are possible.***  1. By my midwife or gynecologist/family doctor  2. Educational program via media such as TV/Radio  3. Health information through work/school  4. Information brochure by mail  5. Through the Internet, social media  6. Other (please specify)………………… |

**Thank you for your participation!**
