# Supplementary material for: Barriers to and Facilitators of Compliance with Clinic-Based Cervical Cancer Screening: Population-Based Cohort Study of Women Aged 23-60 Years
Source: PLoS One. 2015 May 26;10(5):e0128270. doi: 10.1371/journal.pone.0128270 (PMC4444356; doi:10.1371/journal.pone.0128270)
Supplement: S1 Table — (DOCX) [file pone.0128270.s002.docx]

| Population | Study sample | Swedish  female population^a^ |  | Significance |  | Stockholm  County |  | Significance |  |  |  |
| --- | --- | --- | --- | --- | --- | --- | --- | --- | --- | --- | --- |
| Number | 1 510 | 2 734 363 |  |  |  | 641 222 |  |  |  |  |  |
| Employed^a^ | 78.5 | 76.8 |  | NS |  | 77.3 |  | NS |  |  |  |
| Unemployed^a^ | 3 | 7.7 |  | p<.01 |  | 6.9 |  | p<.01 |  |  |  |
| Sick leave and retired^b^ | 2.4 | 7.6 |  | p<.01 |  | 6* |  | p<.01 |  |  |  |

**S1 Table. Difference in socio-demographic variables.**

NS= NS=NOT Significant at 99% confidence interval (2-tailed)
a) Data on employment and unemployment among female population aged 20-64 year from Eurostat (Available in English at <http://epp.eurostat.ec.europa.eu/portal/page/portal/statistics/search_database>)and Swedish Official Data (SCB) (Available in English at [www.scb.se](http://www.scb.se)) and from Swedish Official Data (SCB) (Available in English at [www.scb.se](http://www.scb.se)).
b) Data on sickness compensation/activity among the female population aged 20-64 from Swedish Official Data (SCB) (Available in English at [www.scb.se](http://www.scb.se)). * Data calculated on 641 222 females aged 20-64 year 2012 taken from Swedish Official Data (SCB) (Available in English at [www.scb.se](http://www.scb.se)).
