# Supplementary material for: Barriers to and Facilitators of Compliance with Clinic-Based Cervical Cancer Screening: Population-Based Cohort Study of Women Aged 23-60 Years
Source: PLoS One. 2015 May 26;10(5):e0128270. doi: 10.1371/journal.pone.0128270 (PMC4444356; doi:10.1371/journal.pone.0128270)
Supplement: S2 Table — (DOCX) [file pone.0128270.s003.docx]

**S2 Table. Difference in educational level and income distribution.**

|  | Study Sample | Swedish female  population^a^ | Significance | Stockholm  County | Significance |
| --- | --- | --- | --- | --- | --- |
|  | Nr:1 510 | Nr:2 300 620 |  | Nr:554 799 |  |
| Educational level^a^ |  |  |  |  |  |
| <High school | 3.9 | 10.4 | p<.01 | 9.7 | p<.01 |
| High school or equal | 32.8 | 42.6 | p<.01 | 36 | p<.01 |
| >High school | 63.3 | 45.5 | p<.01 | 52.1 | p<.01 |
| Gross annual income (€)^b^ | Nr:1 510 | Nr:2 734 363 |  | Nr:641 222 |  |
| <13 783 | 9.2 | 17.7 | p<.01 | 16.6 | p<.01 |
| 13 784-27 568 | 13.4 | 27.1 | p<.01 | 22 | p<.01 |
| 27 569-41 353 | 31.6 | 36.8 | p<.01 | 31.8 | NS |
| 41 354-55 137 | 22.5 | 13.2 | p<.01 | 18.7 | p<.01 |
| 55 138-and more | 23.3 | 5.4 | p<.01 | 10.9 | p<.01 |

NS= NS=NOT Significant at 99% confidence interval (2-tailed)
a) Data on educational level among female population aged 23-60 from Swedish Official Data (SCB) (Available in English at [www.scb.se](http://www.scb.se)).
b) Data on income distribution among female population age 20-64 from Swedish Official Data (SCB) (Available in English at [www.scb.se](http://www.scb.se)).
