# Supplementary material for: Barriers to and Facilitators of Compliance with Clinic-Based Cervical Cancer Screening: Population-Based Cohort Study of Women Aged 23-60 Years
Source: PLoS One. 2015 May 26;10(5):e0128270. doi: 10.1371/journal.pone.0128270 (PMC4444356; doi:10.1371/journal.pone.0128270)
Supplement: S3 Table — (DOCX) [file pone.0128270.s004.docx]

**S3. Difference in educational level and income distribution.**

|  | Study Sample Danderyd | Danderyd^a^ | Significant | Study Sample Nacka | Nacka^a^ | Significant | Study Sample Sundbyberg | Sundbyberg^a^ | Significant | | Study Sample  Stockholm City^a^ | Stockholm City^a^ | Significant | Study Sample Värmdö | Värmdö^a^ | Significant |
| --- | --- | --- | --- | --- | --- | --- | --- | --- | --- | --- | --- | --- | --- | --- | --- | --- |
|  | Nr:115 | Nr 7 518 |  | Nr 388 | Nr 24 342 |  | Nr 431 | Nr 11 713 |  | Nr 313 | | Nr 252 221 |  | Nr 263 | Nr 10 047 |  |
| Educational level^a^ |  |  |  |  |  |  |  |  |  |  | |  |  |  |  |  |
| <High school | 6.1 | 3 | NS | 5.4 | 7 | NS | 3.2 | 12 | p<.01 | 1.3 | | 9 | p<.01 | 4.6 | 8 | NS |
| High school or equal | 27.8 | 22 | NS | 32 | 33 | NS | 35.7 | 37 | p<.01 | 27.5 | | 30 | NS | 38.1 | 46 | p<.01 |
| >High school | 66.1 | 73 | NS | 62.6 | 58 | p<.01 | 61 | 50 | p<.01 | 71.2 | | 60 | p<.01 | 57.4 | 48 | p<.01 |
| Gross annual income (€)^b^ | Nr:115 | Nr:7 585 |  | Nr:388 | Nr:25 154 |  | Nr:431 | Nr:12 108 |  | Nr:313 | | Nr:260 179 |  | Nr:263 | Nr:10 572 |  |
| <13 783 | 9.6 | 15.4 | NS | 11.6 | 13.6 | NS | 6.5 | 16.2 | p<.01 | 9.3 | | 17.5 | p<.01 | 9.9 | 12.7 | NS |
| 13 784-27 568 | 10.4 | 15.8 | NS | 14.2 | 19 | p<.01 | 13 | 22.4 | p<.01 | 12.1 | | 20.5 | p<.01 | 15.6 | 22.2 | p<.01 |
| 27 569-41 353 | 31.3 | 21.1 | NS | 24.2 | 29.9 | p<.01 | 38.3 | 33.8 | NS | 34.8 | | 30.1 | NS | 27.8 | 34.7 | NS |
| 41 354-55 137 | 17.4 | 19.5 | NS | 36.9 | 21.2 | p<.01 | 22.7 | 18.8 | NS | 22.7 | | 20 | NS | 25.5 | 19.7 | NS |
| 55 138 and more | 31.3 | 28.2 | NS | 13.1 | 16.3 | NS | 19.5 | 8.8 | p<.01 | 21.2 | | 12 | p<.01 | 21.3 | 10.7 | p<.01 |

NS=NOT Significant at 99% confidence interval (2-tailed)

1. Statistics regarding education level for each municipality are from 2013 for females 25-64 years from Swedish Official Data (SCB) (Available in English at [www.scb.se](http://www.scb.se)).
2. Data on income distribution among female population age 20-64 from Swedish Official Data (SCB) (Available in English at [www.scb.se](http://www.scb.se)).
